# Production of HIV-1 *vif* mRNA Is Modulated by Natural Nucleotide Variations and SLSA1 RNA Structure in SA1D2prox Genomic Region

**DOI:** 10.3389/fmicb.2017.02542

**Published:** 2017-12-18

**Authors:** Masako Nomaguchi, Naoya Doi, Tomoya Yoshida, Takaaki Koma, Shun Adachi, Hirotaka Ode, Yasumasa Iwatani, Masaru Yokoyama, Hironori Sato, Akio Adachi

**Affiliations:** ^1^Department of Microbiology, Graduate School of Medical Science, Tokushima University, Tokushima, Japan; ^2^Department of Infectious Diseases and Immunology, Clinical Research Center, National Hospital Organization Nagoya Medical Center, Nagoya, Japan; ^3^Laboratory of Viral Genomics, Pathogen Genomics Center, National Institute of Infectious Diseases, Tokyo, Japan

**Keywords:** HIV-1, SA1, SLSA1, *vif* mRNA, nNSV, secondary RNA structure, SA1D2prox

## Abstract

Genomic RNA of HIV-1 contains localized structures critical for viral replication. Its structural analysis has demonstrated a stem-loop structure, SLSA1, in a nearby region of HIV-1 genomic splicing acceptor 1 (SA1). We have previously shown that the expression level of *vif* mRNA is considerably altered by some natural single-nucleotide variations (nSNVs) clustering in SLSA1 structure. In this study, besides eleven nSNVs previously identified by us, we totally found nine new nSNVs in the SLSA1-containing sequence from SA1, splicing donor 2, and through to the start codon of Vif that significantly affect the *vif* mRNA level, and designated the sequence SA1D2prox (142 nucleotides for HIV-1 NL4-3). We then examined by extensive variant and mutagenesis analyses how SA1D2prox sequence and SLSA1 secondary structure are related to *vif* mRNA level. While the secondary structure and stability of SLSA1 was largely changed by nSNVs and artificial mutations introduced to restore the original NL4-3 form from altered ones by nSNVs, no clear association of the two SLSA1 properties with *vif* mRNA level was observed. In contrast, when naturally occurring SA1D2prox sequences that contain multiple nSNVs were examined, we attained significant inverse correlation between the *vif* level and SLSA1 stability. These results may suggest that SA1D2prox sequence adapts over time, and also that the altered SA1D2prox sequence, SLSA1 stability, and *vif* level are mutually related. In total, we show here that the entire SA1D2prox sequence and SLSA1 stability critically contribute to the modulation of *vif* mRNA level.

## Introduction

RNAs participate in various cellular processes as mRNAs coding proteins and also as non-coding RNAs involved in regulation of intracellular gene expression, such as micro RNAs and long non-coding RNAs. Single-stranded RNA molecules contain complex secondary/tertiary structures, such as hairpins and stem-loops, which are formed by base-paired and -unpaired nucleotides within sequences of the molecules. Recent advances in RNA analysis have revealed that RNA secondary structures of coding and non-coding RNAs play functional and regulatory roles in various cellular events (Wan et al., 2011; Bevilacqua et al., 2016; Lu and Chang, 2016; Silverman et al., 2016).

Structures crucial for viral replication are indeed found in viral single-stranded RNA genomes like well-known internal ribosomal entry (Whelan, 2013) and packaging signal (Hunter, 2013) sites. HIV-1 genome justly contains numerous functional RNA structures required for viral growth, such as Rev-responsive element, RNA structure involved in *gag-pol* frameshifting, and 5′ leader of RNA genome including *trans*-activation element, splicing donor 1 (SD1, a major donor site), primer binding site, polyadenylation signal, and packaging signal (Muesing et al., 1987; Hauber and Cullen, 1988; Berkhout et al., 1989; Dayton et al., 1989; Olsen et al., 1990; Bartel et al., 1991; Parkin et al., 1992; Keane et al., 2015; Keane and Summers, 2016). Recently, HIV-1 RNA (NL4-3) has been clarified for its entire secondary structure by a chemical-based probing analysis of purified virion RNA (Watts et al., 2009; Pollom et al., 2013). These studies have suggested that there may be RNA structures with unidentified functions in the HIV-1 replication process. In fact, the two works consistently demonstrated that a region surrounding and containing the splicing acceptor 1 (SA1) forms a stem-loop structure, named SLSA1 (Watts et al., 2009; Pollom et al., 2013).

**FIGURE 1:**
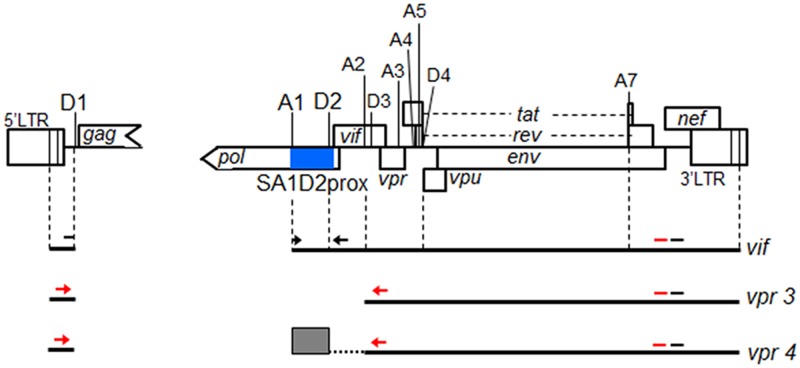
Schematic representation of HIV-1 NL4-3 genome. Various splicing donor (SD) and splicing acceptor (SA) sites in HIV-1 genome are indicated. SA4b, a, c sites are omitted. A blue box indicates SA1D2prox. Black lines represent 4 kb mRNAs of *vif* and *vpr*, and exon2 generated by splicing at SA1 and SD2 is indicated by a gray box. Black arrows and black bars represent amplified regions by qRT-PCR of *vif* mRNA and all HIV-1 mRNAs, respectively. Regions amplified by semiquantitative PCR to analyze *vpr* mRNAs and all HIV-1 mRNAs are shown by red arrows and red bars, respectively.

HIV-1 Vif (virion infectivity factor) protein antagonizes intrinsic retroviral restriction factors, APOBEC3G/F. Thus, the expression and function of Vif are crucial for HIV-1 replication in natural target cells such as CD4^+^-T cells and macrophages (Kirchhoff, 2010; Blanco-Melo et al., 2012; Harris et al., 2012; Malim and Bieniasz, 2012). HIV-1 mRNA species are produced through alternative splicing to express various viral proteins (Schwartz et al., 1990a,b; Purcell and Martin, 1993; Amendt et al., 1995). It has been shown that SDs, SAs, and splicing regulatory elements in viral RNA sequence are involved in this process (Caputi, 2011; Karn and Stoltzfus, 2012). While more than 50 transcripts are produced by alternative splicing, which utilize different combinations of 4 SDs and 10 SAs (Emery et al., 2017) (**Figure 1**), *vif* mRNA is generated through direct splicing between SD1 and SA1 (Schwartz et al., 1990a,b; Purcell and Martin, 1993; Amendt et al., 1995). We have previously demonstrated that *vif* expression level is significantly influenced by some naturally occurring single-nucleotide variations (nSNVs) in the region proximal to SA1 (SA1prox) containing SLSA1, and that most of nSNVs thus identified were clustered in SLSA1 within SA1prox (Nomaguchi et al., 2014, 2016). We have also showed that *vif* expression level is altered by an nSNV at a splicing regulatory element (G_I2_-1) (Widera et al., 2013) just upstream of the Vif start codon (Nomaguchi et al., 2016). These results raised a possibility that more nSNVs with effects on *vif* production may exist around the SA1prox region, and that SLSA1 structure may participate in modulation of *vif* expression. In this study, we identified new nSNVs that significantly affect *vif* level within the sequence from SA1, SD2, and through to the start codon of Vif (designated SA1D2prox) (**Figure 1**). We further investigated the SA1D2prox sequence and SLSA1 secondary structure by extensive functional and predictive analyses on their variants and mutants to gain an insight into their virological relevance and significance for modulation of *vif* production.

## Materials and Methods

### HIV-1 Sequence Analysis

Nucleotide sequences of SA1D2prox were obtained from the HIV-1 sequence database (Los Alamos National Laboratory^1^) using “subtype B” and “one sequence from one patient” option. The 2885 sequences of the region corresponding to nucleotides 4891–5040 of pNL4-3 (GenBank accession number AF324493) (Adachi et al., 1986) were analyzed, excluding sequences containing mixed nucleotides (N, Y, and R) or insertion/deletion. Conservation degree among nucleotide sequences of SA1D2prox was determined by WebLogo3 software^2^ (Schneider and Stephens, 1990; Crooks et al., 2004).

### Construction of Proviral Clones

Proviral clone pNL4-3 (Adachi et al., 1986) was used as a parental clone in the present study. A proviral clone that carries a major consensus sequence of SA1D2prox in HIV-1 subtype B strains was constructed by introduction of three nSNVs (at amino acid positions V234Lctt, L241ctt, and I268att in Pol-integrase) into corresponding sites of pNL4-3, and a resultant clone was designated pNL-pC3. Based on comparative analysis of nucleotide sequences of SA1D2prox, one or two of nSNVs at each amino acid site of Pol-integrase were selected and introduced into pNL4-3. To generate proviral clones that harbor full-length viral genomic SA1D2prox sequences, HIV-1 strains were selected from the HIV-1 compendium 2010-2015 as follows: NL-pSE (SE600057, GenBank accession number KP411828), NL-p04AR (B.AR.04.04AR143170, GenBank accession number DQ383750), NL-p11807 (B.IN.x.11807, GenBank accession number EF694037), NL-pCu19 (B.CU.99.Cu19, GenBank accession number AY586542), NL-pDEMB12 (B.JP.12.DEMB12JP001, GenBank accession number KF716498), NL-pEC003 (B.EC.89.EC003, GenBank accession number AY173959), NL-pMM45 (B.GB.05.MM45d87_GN1, GenBank accession number HM586211), NL-p01TT (B.TT.01.01TT_CRC50069, GenBank accession number EU839608), NL-pCY165 (B.CY.06.CY165, GenBank accession number FJ388947). These nine clones were constructed by introducing multiple nSNVs found within viral genomic SA1D2prox sequences into corresponding sites of pNL4-3 and pNL-pC3. Introduction of nSNVs into pNL4-3 and pNL-pC3 was carried out using PrimeSTAR Max DNA polymerase (Takara Bio).

### Cells, Transfection, and Virus Replication Assay

A human embryonic kidney cell line, HEK 293T (Lebkowski et al., 1985), was cultured in minimal essential medium supplemented with 10% heat-inactivated fetal bovine serum. A human lymphocyte cell line CEM-SS was cultured in RPMI 1640 supplemented with 10% heat-inactivated fetal bovine serum. Transfection of proviral clones into 293T cells were carried out using Lipofectamine 2000 (Thermo Fisher Scientific) as described previously (Nomaguchi et al., 2014, 2016). Virion-associated reverse transcriptase activity was measured as previously described (Willey et al., 1988; Nomaguchi et al., 2013). Equal units of reverse transcriptase activity (10^4^) were inoculated into CEM-SS cells (10^5^) for replication analysis of HIV-1 NL4-3 and its variant clones, and cell culture supernatants were harvested every 3 days. Virus replication was monitored by reverse transcriptase activity released into the culture supernatants.

### Quantitative Reverse Transcription (qRT)-PCR Analysis

293T cells were transfected with proviral clones, and at 20 h post-transfection, total RNA was isolated by using RNeasy Plus Mini kit (Qiagen). Total RNA samples were treated with DNase I (Takara Bio). The nSNVs that affect *vif* expression level were screened with QuantiTect probe RT-PCR kit (Qiagen) using DNase I-treated total RNA as previously described (Nomaguchi et al., 2016). For analysis of *vif* mRNA expression, cDNA was synthesized with Superscript III first-strand synthesis system (Thermo Fisher Scientific) using DNase-I treated total RNA samples and an oligo (dT) primer. The cDNA samples were then subjected to qRT-PCR analysis using Power SYBR Green Master Mix (Thermo Fisher Scientific). Primer sets used for qRT-PCR analysis were as follows: NL-U-F and NL-U-R for all HIV-1 mRNA species (**Figure 1**) (Nomaguchi et al., 2016), NL-D1A1 (Exline et al., 2008; Nomaguchi et al., 2016) and Vif-qPCR (ACCTGCCATCTGTTTTCCATA) for *vif* mRNA (**Figure 1**), Hs-GAPDH-F and Hs-GAPDH-R (Nomaguchi et al., 2016) for GAPDH mRNA. Linearized pNL4-3, pNL-vif+T, and pGAPDH+T vectors were used as standards for all HIV-1 mRNA species, *vif* mRNA, and GAPDH mRNA, respectively, as described previously (Nomaguchi et al., 2016). Expression levels of all HIV-1 mRNA species were used for normalization of transfection efficiency (Nomaguchi et al., 2016).

### Semiquantitative PCR Analysis

Semiquantitative PCR analysis was carried out as previously described using cDNA samples described above (Nomaguchi et al., 2016). Briefly, PCR products were made by using TaKaRa LA Taq Hot Start Version (Takara Bio) and a specific primer set for *vpr* mRNAs (**Figure 1**). As controls, all HIV-1 mRNA species and GAPDH mRNA were amplified using specific primer sets (**Figure 1**) (Nomaguchi et al., 2016) in parallel with *vpr* mRNAs. PCR products were separated on gels prepared by MetaPhor agarose (Lonza), and stained with ethidium bromide. Signal intensities of PCR products were measured by Amersham Imager 600 instrument (GE Healthcare).

### SLSA1 RNA Structure Prediction

SLSA1 RNA structures were predicted by the mfold program^3^ (Zuker, 2003) as described previously (Nomaguchi et al., 2016).

### Statistical Analysis

To evaluate the relationship between *vif* level and SLSA1 structural stability (*dd*G), scatter diagrams with an exponential trendline and a coefficient of determination (*R*^2^) were generated. Multiple correlation coefficients obtained by regression analysis were used to calculate statistical significance of the *R*^2^ values (*F*-test).

### Western Blot Analysis

Proviral clones were transfected into 293T cells, and on day 1 post-transfection, cell lysates were prepared with 1 × TNE buffer (Nomaguchi et al., 2014, 2016). Western blot analysis was carried out as previously described using anti-Vif [anti-1Q (Akari et al., 1999), anti-HIV-1 HXB2 Vif (catalog no. 2221; NIH Research and References Reagent Program) or anti-HIV-1 Vif 319 (catalog no. ab66643; Abcam)] and anti-β-actin clone AC-15 (Sigma–Aldrich) antibodies (Nomaguchi et al., 2014, 2016).

## Results

### Novel nSNVs That Significantly Alter *vif* Expression Level Are Identified within SA1D2prox Region

**FIGURE 2:**
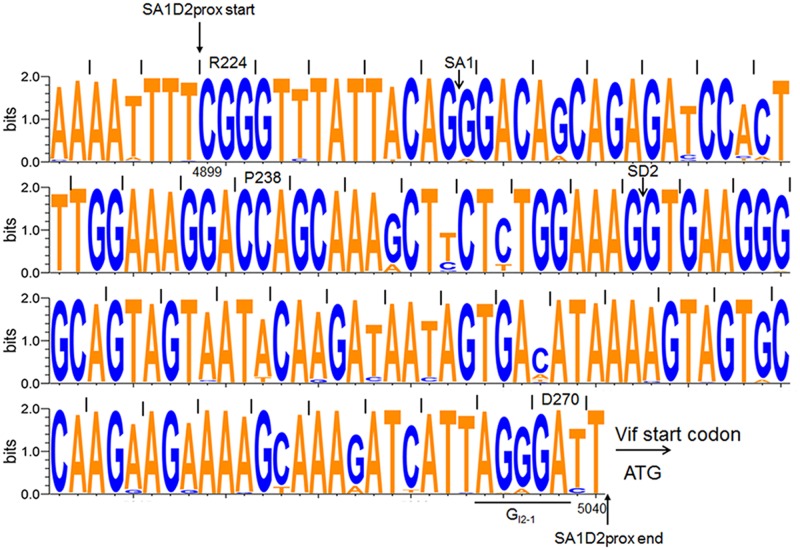
Sequence alignment of SA1D2prox among HIV-1 subtype B viruses. Nucleotide sequence logo was created by WebLogo3 software (http://weblogo.threeplusone.com/create.cgi) (Schneider and Stephens, 1990; Crooks et al., 2004). Sequences (2885 sequences corresponding to nucleotides 4891–5040 of HIV-1 NL4-3) of HIV-1 subtype B were obtained from the HIV-1 sequence database (Los Alamos National Laboratory, http://www.hiv.lanl.gov/). Start and end positions of SA1D2prox region, SA1 and SD2 sites, the start codon for Vif, and a splicing regulatory element G_I2_-1 are indicated. Amino acids and their numbers of NL4-3 Pol-integrase are shown above the sequence. Numbers below the sequence represent nucleotide numbers of HIV-1 NL4-3.

**FIGURE 3:**
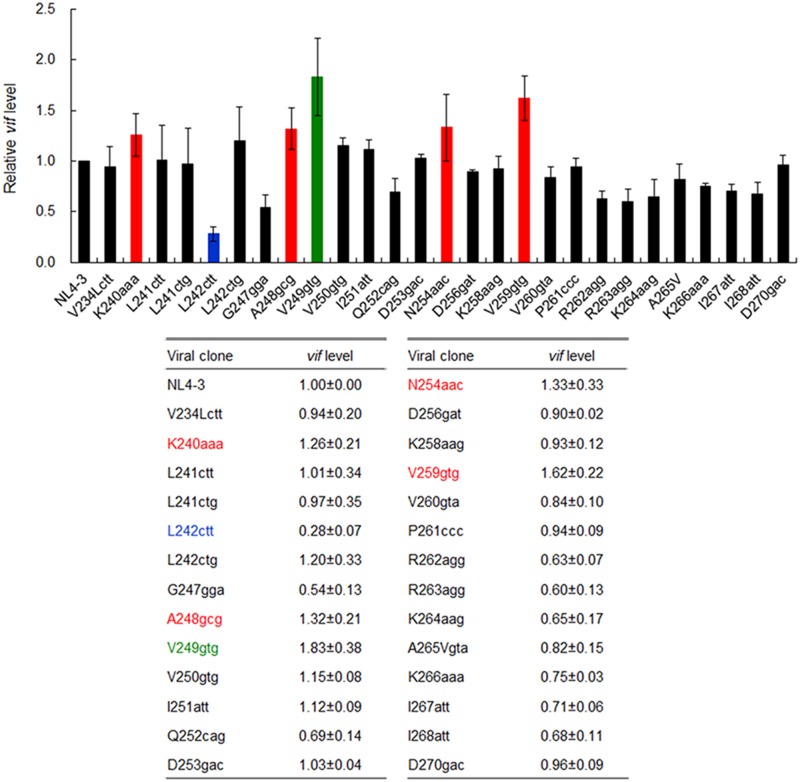
Effects of nSNVs in SA1D2prox on *vif* expression. Proviral clones indicated were transfected into 293T cells, and at 20 h post-transfection, total RNAs were prepared. After DNase I-treatment, samples were subjected to qRT-PCR analysis by a specific primer set for *vif* transcript (**Figure 1**). For clones designated K240aaa, L242ctt, A248gcg, V249gtg, N254aac, and V259gtg, cDNA samples synthesized using DNase I-treated RNA and oligo (dT) primer were subjected to qRT-PCR analysis. Expression levels of all HIV-1 transcripts/mRNAs and GAPDH transcript/mRNA were analyzed by qRT-PCR in parallel as transfection and internal controls, respectively. A *vif* expression level in each sample was normalized by those of all HIV-1 transcript/mRNA species and GAPDH transcript/mRNA. *Vif* expression levels relative to that by NL4-3 are presented. Blue letters/bar, red letters/bars, and green letters/bar indicate low, high, and excessive *vif* types (Nomaguchi et al., 2016), respectively. Mean values ± SD from four independent experiments are shown.

We previously showed that some nSNVs, found by comparison of HIV-1/SIVcpz sequences in SA1prox region (corresponding to amino acids R224 to P238 of HIV-1 NL4-3 Pol-integrase in **Figure 2**), modulate *vif* expression level and thereby alter viral replication potential (Nomaguchi et al., 2014, 2016). We also showed in that study (Nomaguchi et al., 2016) that an nSNV affecting *vif* production is present at the splicing regulatory element G_I2_-1 (Widera et al., 2013). We here asked whether such nSNVs still more exist in adjacent to SA1prox (Nomaguchi et al., 2016). Nucleotide variations were searched for SA1D2prox region (nucleotides 4899–5040 of HIV-1 NL4-3 in **Figures 1**, **2**) by aligning sequences of HIV-1 subtype B strains deposited in the database (Los Alamos National Laboratory^4^). As seen in **Figure 2**, while nucleotide sequences are fairly well conserved, there are a number of sporadic variations. We noted that many of them are synonymous variations, probably being due to constraints on the amino acid sequence of Pol-integrase to maintain its function. Based on this analysis of SA1D2prox, one or two nSNVs at each codon excluding those in SA1prox were chosen, and were introduced into an HIV-1 proviral clone NL4-3 (**Figure 3**). A variant V234Lctt was also constructed, because Lctt is a major sequence at the site among HIV-1 subtype B strains (**Figure 2**). Thus, a total of 27 proviral clones were newly constructed.

In order to identify new nSNVs in SA1D2prox that alter *vif* expression level, we performed qRT-PCR analyses. Proviral clones constructed were transfected into 293T cells in parallel with a parental NL4-3 clone. At 20 h post-transfection, DNase I-treated total RNA samples were prepared and subjected to qRT-PCR analysis using a specific primer set for *vif* transcript. Proviral clones that could affect *vif* mRNA production were further analyzed by qRT-PCR with cDNA synthesized using an oligo (dT) primer and DNase I-treated total RNA as a template. Predictably, essentially the same results were obtained for levels of the *vif* transcript and *vif* mRNA. As shown in **Figure 3**, *vif* levels by most clones tested were similar or modestly changed relative to that by NL4-3. Because viral replication potentials are empirically known not to be substantially influenced by such small changes (Nomaguchi et al., 2016), we selected six variants for further analysis, which carry an nSNV (K240aaa, L242ctt, A248gcg, V249gtg, N254aac, or V259gtg) that exhibit *vif* expression levels under 0.5 or over 1.2 relative to NL4-3 (colored clones in **Figure 3**). Expression levels of Vif protein by these variants fluctuated in a consistent manner with *vif* mRNA levels as expected (data not shown) (Nomaguchi et al., 2016).

**FIGURE 4:**
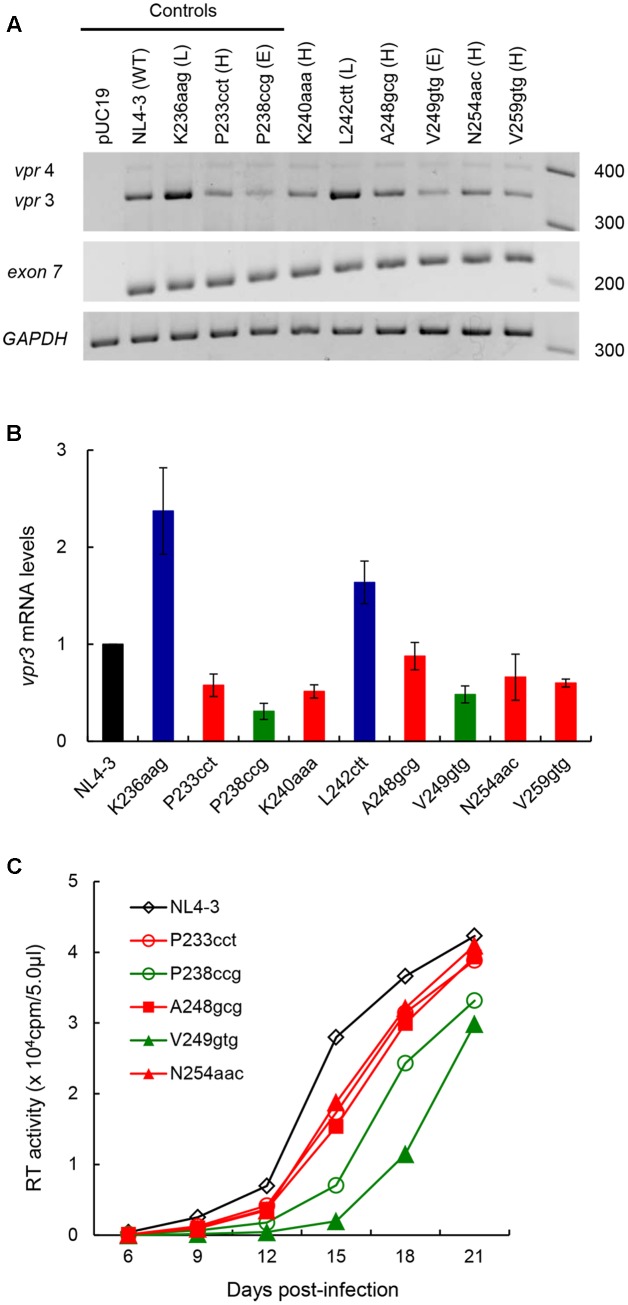
Effects of *vif*-affecting nSNVs on *vpr* expression and virus replication. **(A)** *Vpr* expression pattern. Semiquantitative PCR was carried out using a specific primer set for *vpr* mRNAs (**Figure 1**), and cDNA samples were prepared as described in **Figure 3**. NL4-3 and K236aag/P233cct/P238ccg clones were used as a parental clone and as standard control clones for low (L)/high (H)/excessive (E) *vif* types (Nomaguchi et al., 2016), respectively. Expression levels of all HIV-1 mRNA species (exon7) and GAPDH mRNA were analyzed by semiquantitative PCR in parallel as transfection and internal controls, respectively. RNA size markers in nucleotides are indicated on the right. Representative data from four independent experiments are shown. **(B)** Quantification of *vpr* expression level. Signal intensities of semiquantitative RT-PCR products were quantitated by Amersham Imager 600 instrument. Intensity of *vpr3* mRNA in each sample was normalized by those of all HIV-1 mRNA species (exon 7) and GAPDH mRNA. Normalized mRNA intensity in each sample relative to that of NL4-3 is presented. Mean values ± SD from four independent experiments are shown. **(C)** Viral growth kinetics in CEM-SS cells. Viruses were prepared from 293T cells transfected with proviral clones indicated, and equal units of reverse transcriptase activity (10^4^) were inoculated into CEM-SS cells (10^5^). Virus replication was monitored by reverse transcriptase activity released into the culture supernatants. The results shown were reproduced in another independent experiment.

We previously showed that proviral clones carrying an nSNV in SA1prox can be categorized into three types based on their *vif* and Vif expression levels, i.e., low, high, and excessive *vif* types (Nomaguchi et al., 2016). These types are also distinguished and characterized by inverse correlation of *vif* and *vpr* expression levels, and by viral growth capacity in the presence and absence of APOBEC3G (Nomaguchi et al., 2016). To further substantiate this virological observation, and to classify and characterize newly constructed six clones (K240aaa, L242ctt, A248gcg, V249gtg, N254aac, and V259gtg in **Figure 3**), we first examined effects of these nSNVs on *vpr* mRNA production by semiquantitative PCR analysis as previously described (Nomaguchi et al., 2016). Proviral clones were transfected into 293T cells in parallel with control clones [parental clone (NL4-3), low *vif* type (K236aag), high *vif* type (P233cct), and excessive *vif* type (P238ccg) in Nomaguchi et al., 2016]. At 20 h post-transfection, cells were collected and cDNA samples were prepared as described above. As shown in **Figure 4A**, these clones tested displayed different levels of *vpr* mRNA expression. L242ctt produced *vpr* mRNA at a similar level with K236aag and at a higher level than NL4-3, indicating it belongs to the low *vif* group. *Vpr* levels by the other four (K240aaa, A248gcg, N254aac, and V259gtg) and one (V249gtg) new clones were similar to those by P233cct (high *vif*) and P238ccg (excessive *vif*), respectively. As shown in **Figure 4B**, signal intensities of PCR products in four independent experiments quantitatively confirmed the results for *vpr* mRNA expression levels by L242ctt and two variants (K240aaa and V259gtg) relative to that by NL4-3 in **Figure 4A**. On the other hand, **Figure 4B** gave the results that require further experimentation for A248gcg, V249gtg, and N254aac. Although mean values for *vpr* expression level for A248gcg and N254aac were lower than that for NL4-3, statistically significant difference was not obtained. Another variant V249gtg showed an intermediate *vpr* expression level between those by P233cct (high *vif*) and P238ccg (excessive *vif*). We therefore examined the three variant clones (A248gcg, V249gtg, and N254aac) for their biological consequences by assessing viral growth capacity in CEM-SS cells with a low APOBEC3G level (Nomaguchi et al., 2016). In CEM-SS cells, viruses of the high *vif* type grew moderately poorly relative to NL4-3, and those of the excessive *vif* type propagated further more poorly relative to a virus of the high *vif* type (Nomaguchi et al., 2016). Input viruses were prepared from 293T cells transfected with test (A248gcg, V249gtg, and N254aac) or control (NL4-3, P233cct, and P238ccg) clones, and were inoculated into CEM-SS cells. As shown in **Figure 4C**, A248gcg and N254aac grew markedly similarly with P233cct (high *vif*), slightly more inefficiently than NL4-3. Another test clone V249gtg displayed significantly slow growth kinetics than the others including P238ccg of the excessive *vif* type. This experiment was repeated with similar results. Collectively, based on *vif*/*vpr* expression levels and replication potentials (**Figures 3**, **4**), six new proviral clones were grouped as low (L242ctt), high (K240aaa, A248gcg, N254aac, and V259gtg), and excessive (V249gtg) *vif* type viruses.

### The nSNVs That Affect *vif* Expression Level Are Widely Distributed in the SA1D2prox Region and Many of Them Are Clustered in SLSA1

**FIGURE 5:**
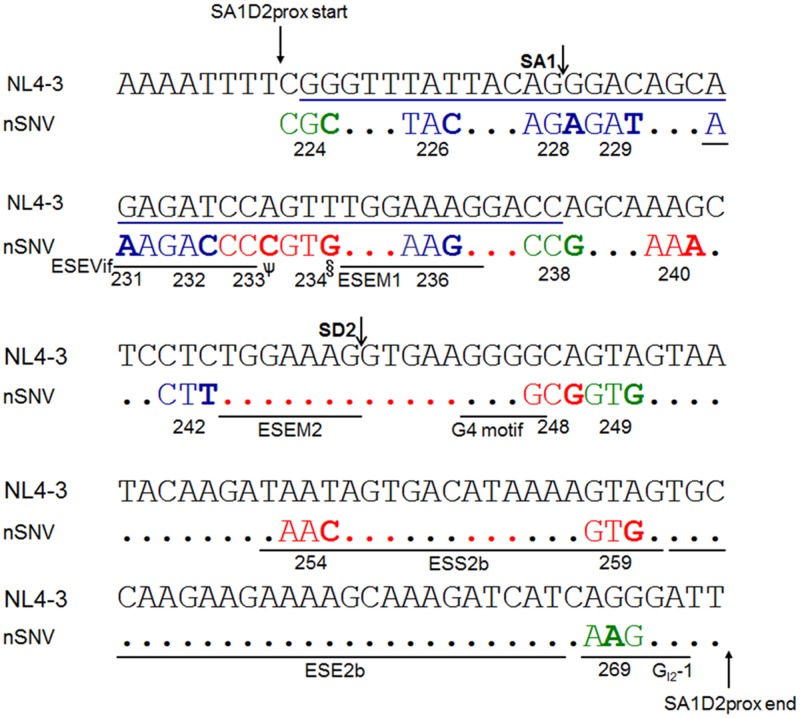
Locations of nSNVs in SA1D2prox sequence (nucleotides 4899-5040 of HIV-1 NL4-3) that significantly alter *vif* expression level. Numbers below the sequences represent amino acid positions of HIV-1 NL4-3 Pol-integrase. SA1/SD2 sites and reported splicing regulatory elements (ESEVif, ESE-M1, ESE-M2, G4 motif, ESS2b, ESE2b, and G_I2_-1 in Kammler et al., 2006; Exline et al., 2008; Mandal et al., 2009; Widera et al., 2013; Brillen et al., 2017) are shown. A blue line indicates the SLSA1 region. The nSNVs that significantly alter *vif* expression level are shown by colored and bold letters. Blue/red/green letters indicate low/high/excessive *vif* types (Nomaguchi et al., 2016), respectively. Red dots show the sites for which no proviral clones for analysis were constructed, because the sequences are highly conserved among HIV-1 subtype B strains. Black dots represent the sites for which proviral clones were constructed and analyzed, but nSNVs there were found to affect *vif* production only modestly. ψ, G, and T variants are also high *vif* type. §, C variant is also high *vif* type.

Several splicing regulatory elements that affect *vif* mRNA production and/or usage of SA1 and SD2 sites have been reported. These elements include, from 5′ to 3′, exonic splicing enhancer Vif (ESEVif), ESE-M1/M2, G4 motif, exonic splicing silencer 2b (ESS2b), ESE2b, and G_I2_-1 (Kammler et al., 2006; Exline et al., 2008; Mandal et al., 2009; Widera et al., 2013; Brillen et al., 2017). As presented in **Figure 5**, we here mapped twenty nSNVs that alter *vif* expression level identified by us so far (results in this study and in Nomaguchi et al., 2016) to SA1D2prox along with above splicing regulatory elements. Of these effective nSNVs, 13 increased and 7 decreased *vif* expression. While found throughout SA1D2prox, many of the nSNVs valid for modulation of *vif* production resided in its 5′ half including SLSA1 (15/20 nSNVs). All *vif*-decreasing nSNVs were located at the 5′ half and only *vif*-increasing nSNVs were present in the 3′ half. Of note, twelve effective nSNVs were clustered in SLSA1, suggesting a possible involvement of SLSA1 (sequence and/or structure) in modulation of *vif* production. Another important point to be mentioned in **Figure 5** is that six nSNVs (V234gtg, P238ccg, K240aaa, L242ctt, A248gcg, and V249gtg) are located at distinct sites from elements reported previously (Kammler et al., 2006; Exline et al., 2008; Mandal et al., 2009; Widera et al., 2013; Brillen et al., 2017). This finding suggests a potential existence of novel splicing regulatory elements so far unidentified. In sum, seventeen codon sites (twenty nucleotides) in SA1D2prox (142 nucleotides) are found to be effective for modulation of *vif* expression level (**Figure 5**). It is clear from these results that SA1D2prox is a critical functional region to modulate *vif* expression.

### Changes in SLSA1 Secondary Structure and Stability by nSNVs Are Not Well-Correlated with Those in *vif* Expression Level

**FIGURE 6:**
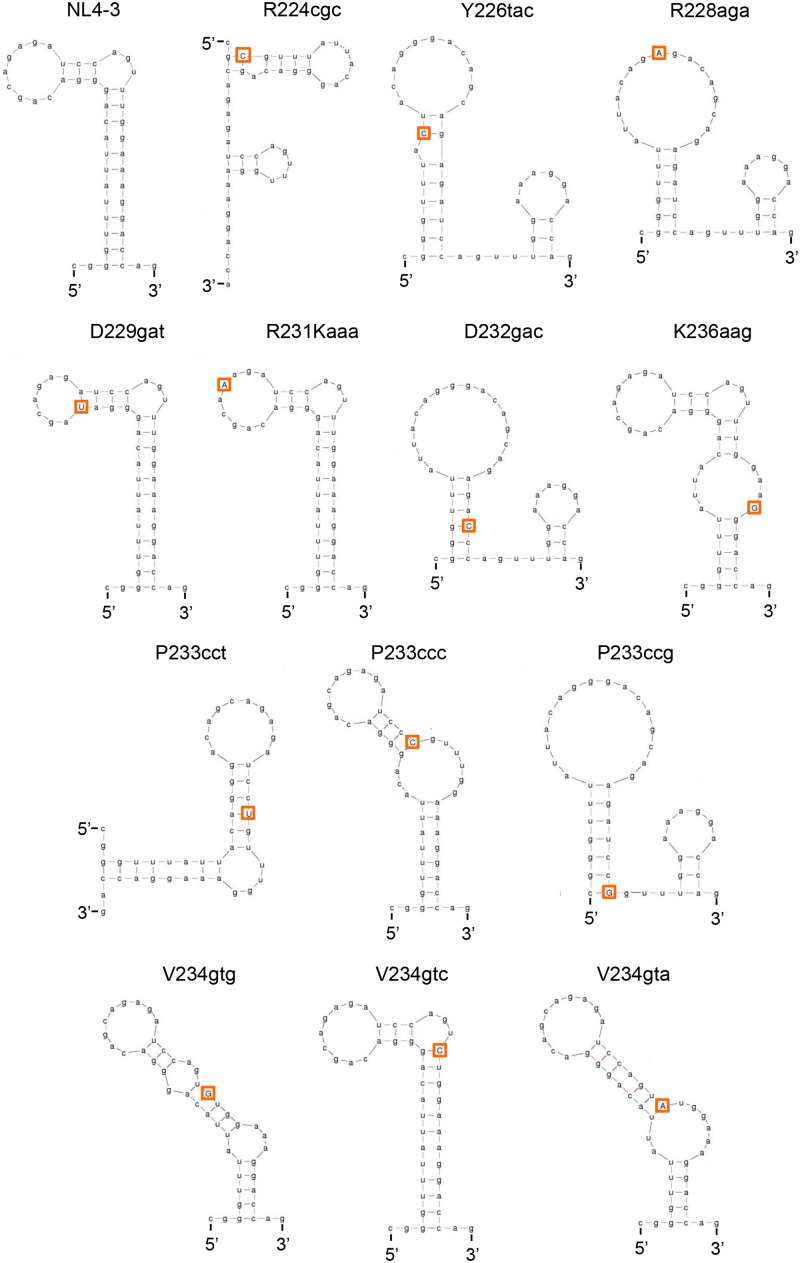
Changes in SLSA1 RNA secondary structure by nSNVs within SLSA1. Various nSNVs analyzed are indicated by orange-boxed capital letters. Secondary RNA structure for the SLSA1 sequence carrying an nSNV was predicted by mfold program (Zuker, 2003).

**FIGURE 7:**
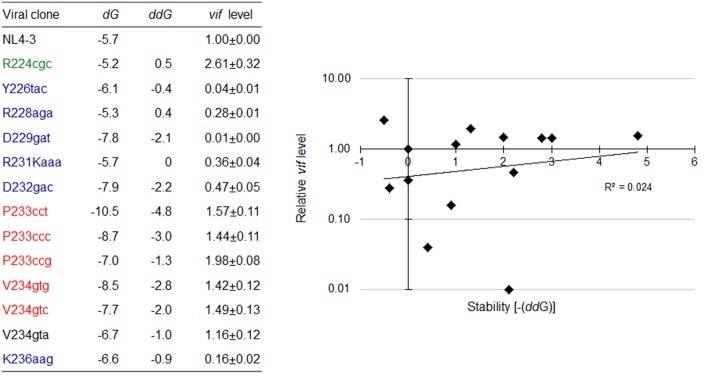
Changes in *vif* expression level and SLSA1 RNA stability by nSNVs within SLSA1. Free energy (*d*G) for the SLSA1 sequence carrying an nSNV was predicted by mfold program (Zuker, 2003). RNA stability (*dd*G) is presented as difference of free energy between each nSNV and NL4-3. For determination of *vif* production level, proviral clones indicated were transfected into 293T cells, and at 20 h post-transfection, DNase I-treated total RNAs were prepared. After synthesis of cDNA using DNase I-treated RNA and oligo (dT) primer, samples were subjected to qRT-PCR analysis using a specific primer set for *vif* mRNA. Expression levels of all HIV-1 mRNA species and GAPDH mRNA were analyzed by qRT-PCR in parallel for transfection and internal controls, respectively. A *vif* expression level in each sample was normalized by those of all HIV-1 mRNA species and GAPDH mRNA. *Vif* expression levels relative to that by NL4-3 are presented. Mean values ± SD from three independent experiments are shown. Scatter diagram on the right was created by plotting *vif* expression levels and SLSA1 stabilities [–(*dd*G)] of variant clones with nSNVs relative to those of NL4-3. Exponential trendline and coefficient of determination (*R*^2^ = 0.024, *p* = 0.549 by *F*-test) are shown. Blue, red, and green letters indicate low, high, and excessive *vif* types (Nomaguchi et al., 2016), respectively. In this figure, the data of relative *vif* expression levels in our previous report (Nomaguchi et al., 2016) were used for clones R224cgc, Y226tac, R228aga, D229gat, R231Kaaa, D232gac, and K236aag for easy comparison.

Accumulating evidence suggests that RNA secondary structures impact mRNA processing such as the polyadenylation, splicing, degradation, and translation (Wan et al., 2011; Bevilacqua et al., 2016; Lu and Chang, 2016; Silverman et al., 2016). Because many of the nSNVs that significantly affect *vif* expression level were localized within SLSA1 (**Figure 5**), we hypothesized that SLSA1 sequence and/or structure may contribute to modulation of *vif* production. In order to examine relationship between *vif* production and SLSA1, we utilized the mfold program (Zuker, 2003) to predict changes in SLSA1 secondary structure and free energy (*d*G) caused by nSNVs in SLSA1. When multiple structures were expected for an nSNV-bearing SLSA1, a structure with lowest *d*G, i.e., the most stabilized structure, was selected. In fact, a structural shape of HIV-1 SLSA1 RNA (NL4-3) inferred in this way was highly similar to that solved by a chemical probing approach (**Figure 6**) (Pollom et al., 2013). We then investigated effects of 13 nSNVs in SLSA1 (R224cgc, Y226tac, R228aga, D229gat, R231Kaaa, D232gac, P233cct/ccc/ccg, V234gtg/gta/gtc, and K236aag in **Figure 5**) on *vif* expression level and RNA shape/stability (**Figures 6**, **7**). Stability shift by individual nSNVs of SLSA1 secondary structure was determined by difference from NL4-3 *d*G as *dd*G. Nine clones designated R224cgc, Y226tac, R228aga, D229gat, R231Kaaa, D232gac, P233cct, V234gtg, and K236aag were previously analyzed for *vif* expression and SLSA1 stability (Nomaguchi et al., 2016). Therefore, to obtain experimental data on *vif* production, six clones, i.e., P233cct/ccc/ccg and V234gtg/gta/gtc were assayed here. *Vif* expression levels were assessed by qRT-PCR analysis using cDNA samples and a specific primer set for *vif* mRNA. As is clear in **Figures 6**, **7**, most of the nSNV variants predictively showed different results of the shape and stability of SLSA1 from those for NL4-3. SLSA1 RNA structure carrying V234gtc was identical to that of NL4-3 (**Figure 6**), whereas the energy of its structure was moderately increased by formation of a G-C canonical pair instead of a G⋅U wobble pair (*d*G is -7.7 for V234gtc and -5.7 for NL4-3) (**Figure 7**). Moreover, V234gtc produced higher level of *vif* mRNA relative to NL4-3 (∼1.5) (**Figure 7**). While only R231Kaaa gave the same SLSA1 structure (**Figure 6**) and energy with those of NL4-3 (*d*G = -5.7) (**Figure 7**), its *vif* expression level was significantly lower than that of NL4-3 (∼0.36) (**Figure 7**), indicating the importance of the nucleotide sequence at this site. To obtain a clue to quantitative relationship of *vif* mRNA and SLSA1, relative *vif* expression levels and SLSA1 stability values of the 13 variants were plotted to a scatter diagram using NL4-3 as a standard (**Figure 7**). No clear correlation was observed between them (*R*^2^ = 0.024).

### *Vif* Expression Levels Fluctuate Independently of Changes in SLSA1 Secondary Structure Induced by nSNVs and Artificial Mutations That Recover the Original Structural Shape of NL4-3 SLSA1

**FIGURE 8:**
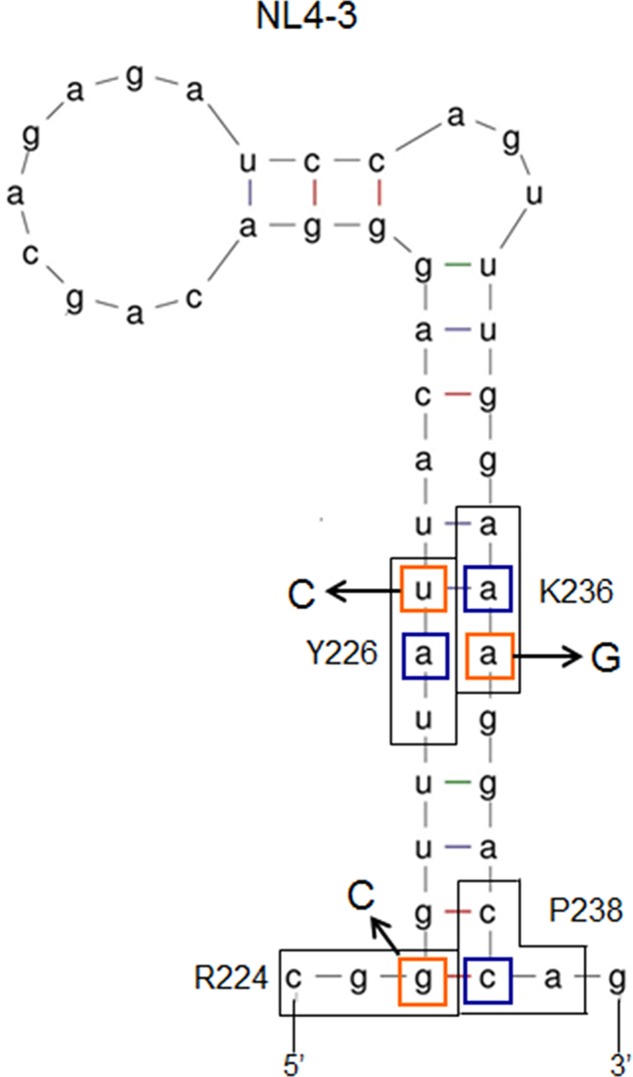
Secondary RNA structure of HIV-1 NL4-3 SLSA1. Secondary RNA structure of NL4-3 SLSA1 was predicted by mfold program (Zuker, 2003). In the structure, amino acid sites R224/Y226/K236/P238 of NL4-3 Pol-integrase, the corresponding codons (black-boxed), nucleotide sites analyzed (orange-boxed), and the complementary mutations (blue-boxed) are shown. Nucleotide substitutions are also shown by arrows.

**FIGURE 9:**
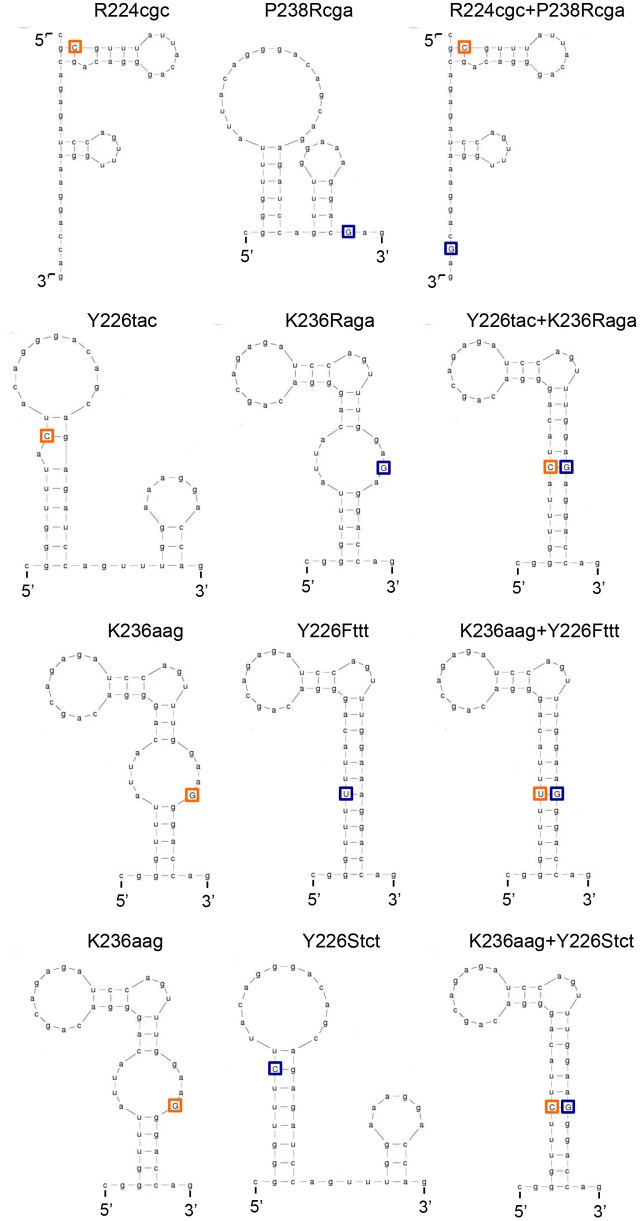
Predicted secondary RNA structures of SLSA1 structural mutants. Secondary RNA structures of the SLSA1 mutants were predicted by mfold program (Zuker, 2003). In the structures of SLSA1 variants, their nSNVs analyzed are indicated by orange-boxed capital letters. Complementary mutations to the nSNVs are indicated by blue-boxed capital letters.

To further investigate relationship between *vif* production and SLSA1 secondary structure, we newly constructed several SLSA1 structural mutants. Since some nSNVs (R224cgc, Y226tac, and K236aag), located at the stem of NL4-3 SLSA1, exhibited relatively strong effects on *vif* expression level and SLSA1 shape (**Figures 6**, **7**) (Nomaguchi et al., 2016), we generated various SLSA1 mutants with the NL4-3 structural shape by introduction of an artificial mutation restoring complementarity of the stem in combinations with the nSNVs (**Figure 8**). A mutation P238Rcga was introduced into R224cgc variant (complementary sites are underlined) to construct a double mutant designated R224cgc+P238Rcga. This SLSA1 double mutant was predicted to have the same SLSA1 shape (*d*G = -5.2) with that of R224cgc variant (**Figure 9**), whereas the NL4-3 shape with a higher energy (*d*G = -4.4) was also expected as one of predictable structures. A mutation K236Raga was introduced into Y226tac variant to construct a double mutant designated Y226tac+K236Raga. This double mutant was predicted to form the same SLSA1 structure with that of NL4-3 (**Figure 9**). For K236aag variant, two SLSA1 structural mutants were constructed by introducing two mutations. One was Y226Fttt that forms a G⋅U wobble base pair, and the other was Y226Stct that forms a G-C canonical base pair. While these double mutants (K236aag+Y226Fttt and K236aag+Y226Stct) were predicted to have the same SLSA1 structural shape with that of NL4-3 (**Figure 9**), free energies for the structures were expected to be different owing to formation of G⋅U or G-C base pair.

**FIGURE 10:**
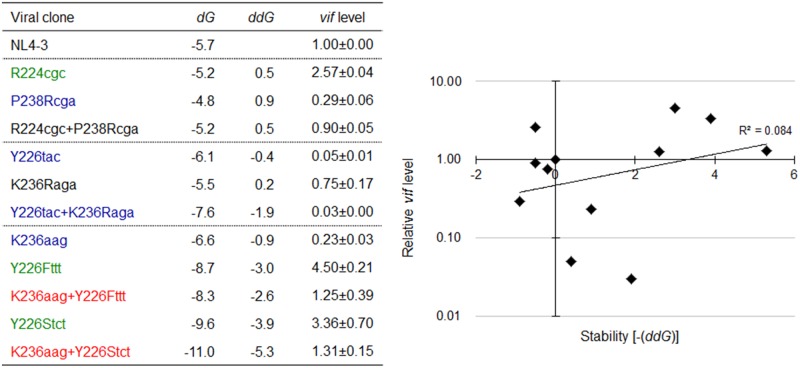
Relationship between the *vif* expression level and SLSA1 RNA stability. SLSA1 variants in **Figure 9** were analyzed for *vif* expressions and SLSA1 stabilities. Free energy (*d*G) for each SLSA1 RNA is predicted by mfold program (Zuker, 2003). Stability (*dd*G) of SLSA1 RNA structure is presented as difference of free energy between mutants and NL4-3. For determination of *vif* production level, proviral clones indicated were transfected into 293T cells, and total RNA was prepared from cells collected at 20 h post-transfection. DNase I-treated RNAs were used for cDNA synthesis with oligo (dT) primer, and resultant cDNA samples were subjected to qRT-PCR analysis using a specific primer set for *vif* mRNA. Expression levels of all HIV-1 mRNA species and GAPDH mRNA were analyzed by qRT-PCR in parallel for transfection and internal controls, respectively. A *vif* expression level in each sample was normalized by those of all HIV-1 mRNA species and GAPDH mRNA. *Vif* expression levels relative to those by NL4-3 are presented. Mean values ± SD from three independent experiments are shown. Scatter diagram on the right was created by plotting *vif* expression levels and SLSA1 stabilities [–(*dd*G)] of the test clones relative to those of NL4-3. Exponential trendline and coefficient of determination (*R*^2^ = 0.084, *p* = 0.161 by *F*-test) are shown. Blue, red, and green letters indicate low, high, and excessive *vif* types (Nomaguchi et al., 2016), respectively.

The above variants or mutants (eleven clones in total) carrying nSNV alone, artificial mutation alone, or double mutations were analyzed for their *vif* expression levels and SLSA1 stabilities. As readily seen in **Figure 10**, all single mutants carrying nSNVs (R224cgc, Y226tac, and K236aag) or artificial mutations (P238Rcga, K236Raga, Y226Fttt, and Y226Stct) showed significantly altered relative *vif* levels. Especially, Y226 mutations (Y226tac, Y226Fttt, and Y226Stct) highly affected *vif* production (values relative to NL4-3 level were 0.05, 4.50, and 3.36, respectively). Y226 site is located at the polypyrimidine tract just upstream of SA1 (**Figure 5**), and thus its mutations may markedly influence the splicing at SA1 through alteration of binding to splicing factors. We have previously shown that HIV-1 strains carrying multiple nSNVs in SA1prox exist in infected individuals, and that such nSNVs exhibit an additive effect on modulation of *vif* production (Nomaguchi et al., 2016). We thus suggested that *vif* expression level was determined by relative strength of splicing regulatory sites around SA1 site (Nomaguchi et al., 2016). In agreement with our previous results, each mutation in the double mutants (R224cgc+P238Rcga, K236aag+Y226Fttt, and K236aag+Y226Stct) appeared to separately affect *vif* production, resulting in similar expression levels to that by NL4-3 (relative levels were from 0.90 to 1.31) (**Figure 10**). These results confirmed independent contribution of individual mutations in SLSA1 to *vif* production. In contrast, remarkably decreased *vif* expression level by Y226tac was not recovered with introduction of K236Raga into Y226tac (Y226tac+K236Raga), supporting that Y226tac exhibits a specially potent effect on *vif* production as described above. To quantitatively evaluate relationship between *vif* production and SLSA1, *vif* levels relative to that by NL4-3 and SLSA1 stabilities of the 11 variants/mutants were plotted to a scatter diagram (**Figure 10**). Clear correlation was not observed (*R*^2^ = 0.084). In total, changes in SLSA1 structural shape/stability by mutations were not significantly correlated with those in *vif* expression (**Figures 9**, **10**). This conclusion was further supported by the data that *vif* expression levels by R224cgc and by R224cgc+P238Rcga were considerably different (approximately threefold difference in *vif* expression level), although the secondary structures and stabilities of SLSA1 RNAs of these two variants were the same each other (**Figures 9**, **10**).

### Inverse Correlation between *vif* Expression Level and SLSA1 Structural Stability Is Observed for Proviral Clones Carrying a Full-Length Viral Genomic SA1D2prox Sequence Found in the HIV-1 Population

**FIGURE 11:**
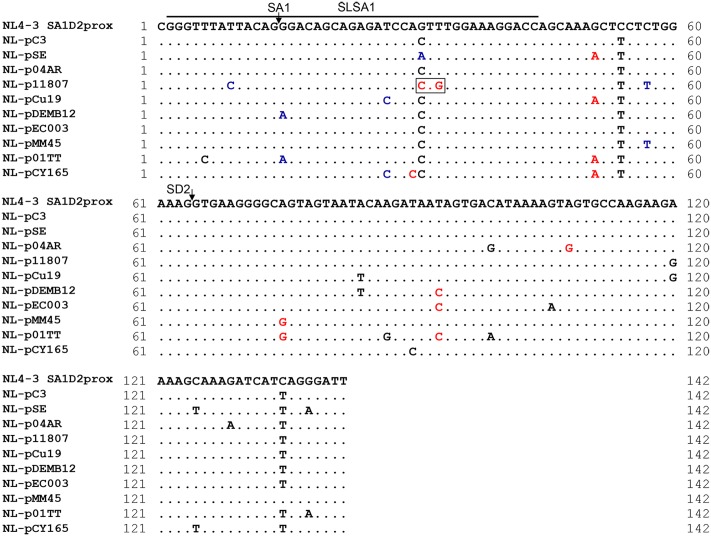
Naturally occurring full-length SA1D2prox sequences analyzed in this study. Full-length SA1D2prox sequences derived from viral strains in the HIV-1 subtype B population are shown. NL-pC3 represents a consensus sequence of SA1D2prox in HIV-1 subtype B strains. SLSA1 region and SA1/SD2 sites are indicated. Blue and red letters show nSNVs that, upon introduction into NL4-3, phenotypically change it to the low and high *vif* types (Nomaguchi et al., 2016), respectively. The nSNVs that moderately affect *vif* expression level are indicated by black letters (*vif* levels were from 0.5 to 1.2 relative to that by NL4-3). V234Lctg site is boxed. This variation contains two and one nucleotide substitutions relative to NL4-3 and NL-pC3, respectively.

As observed in **Figure 2**, there are well-conserved and variable nucleotides in SA1D2prox sequences from HIV-1 subtype B strains. We have shown so far that HIV-1 strains carrying multiple nSNVs in SA1D2prox exist in infected humans, and that each nSNV in the region can independently affect *vif* production to a different degree (**Figures 2**, **3**, **7** and Nomaguchi et al., 2016). Moreover, considering high mutability and adaptability of HIV-1, it is reasonable to assume that mutations may accumulate in SA1D2prox to express *vif* mRNA at a level tolerable for viral replication under a given environment. To test this assumption, we first constructed HIV-1 variants carrying a naturally occurring entire SA1D2prox sequence, and next amply characterized them for various properties. Finally, relationship between *vif* level and SLSA1 stability of variant clones was evaluated by scatter diagram analysis. For this direction, we selected nine SA1D2prox sequences from HIV-1 subtype B strains in the HIV-1 compendium 2010–2015 (**Figure 11**). The naturally occurring SA1D2prox sequences were carefully chosen to contain different combinations of multiple nSNVs that increase or decrease *vif* expression level. We thus constructed nine proviral clones that carry each full-length SA1D2prox sequence from HIV-1 subtype B strains (NL-pSE, NL-p04AR, NL-p11807, NL-pCu19, NL-pDEMB12, NL-pEC003, NL-pMM45, NL-p01TT, and NL-pCY165 in **Figure 11**). In addition, we constructed four proviral clones carrying an unanalyzed nSNV (V234Lctg, D256Egaa, D256Egag, and V259Iata) found in the above nine SA1D2prox sequences. Numerous sequences of subtype B strains in the HIV-1 compendium indicate that HIV-1 NL4-3 has a minor sequence in SA1D2prox at Pol-integrase amino acid codons 234, 241, and 268. We therefore constructed a new proviral clone designated NL-pC3 by introducing three variations (V234Lctt, L241ctt, and I268att) into NL4-3 as a standard with a consensus sequence in SA1D2prox.

**FIGURE 12:**
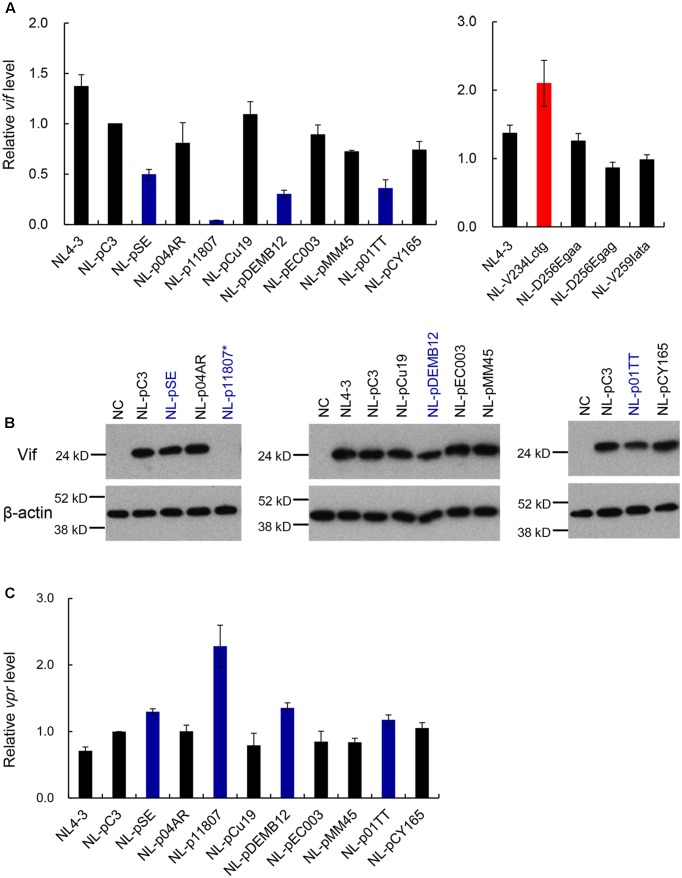
Characterization of proviral clones with a naturally occurring full-length SA1D2prox sequence. Various proviral clones were examined for *vif* mRNA production, Vif expression, and *vpr* mRNA production. **(A)** Expression levels of *vif* mRNAs by various variants. Proviral clones indicated were transfected into 293T cells, and at 20 h post-transfection, cells were collected for extraction of total RNA. DNase I-treated total RNAs were used to synthesize cDNAs with oligo (dT) primer, and the cDNA samples were subjected to qRT-PCR analysis using a specific primer set of *vif* mRNA. Expression levels of all HIV-1 mRNA species and GAPDH mRNA were analyzed by qRT-PCR in parallel for transfection and internal controls, respectively. A *vif* expression level in each sample was normalized by those of all HIV-1 mRNA species and GAPDH mRNA. *Vif* expression levels relative to that by NL-pC3 are presented. Blue and red bars indicate clones that exhibit lower and higher *vif* expression levels, respectively, relative to that by NL-pC3. Mean values ± SD from three independent experiments are shown. All clones were simultaneously assayed for *vif* production, but results obtained are separately shown for clarity. **(B)** Expression of Vif proteins by various variants. 293T cells were transfected with proviral clones indicated. On day 1 post-transfection, cell lysates were prepared, and analyzed by Western blotting using anti-Vif and anti-β-actin antibodies. Migration positions of mass standards are shown on the left. Representative data from at least two independent experiments are shown. Blue letters indicate clones that exhibit lower *vif* expression level relative to that by NL-pC3. NL-p11807^∗^, undetectable. **(C)** Expression levels of *vpr3* mRNA by various variants. Semiquantitative PCR was carried out using cDNA samples prepared as described in **(A)** and a specific primer set for *vpr* mRNAs. Expression levels of all HIV-1 mRNA species and GAPDH mRNA were analyzed by semiquantitative PCR in parallel for transfection and internal controls, respectively. Signal intensities of semiquantitative RT-PCR products were quantitated by Amersham Imager 600 instrument. Intensity of *vpr3* mRNA in each sample was normalized by those of all HIV-1 mRNA species and GAPDH mRNA. Normalized mRNA intensity in each sample relative to that by NL-pC3 is presented. Blue bars show clones with decreased *vif* expression level relative to NL-pC3. Mean values ± SD from three independent experiments are shown.

We then assessed *vif* expression level of the fourteen test and control clones newly constructed. As shown in **Figure 12A**, *vif* level by NL-pC3 was modestly low relative to that by NL4-3, probably due to an effect of I268att (relative level to NL4-3 was ∼0.7 in **Figure 3**). No test clones with a naturally occurring full-length SA1D2prox sequence from different viral strains significantly increased *vif* production level relative to that by NL-pC3. While five out of nine clones (NL-p04AR, NL-pCu19, NL-pEC003, NL-pMM45, and NL-pCY165) produced *vif* mRNA to a similar extent with NL-pC3 (relative levels to NL-pC3 were from 0.72 to 1.09), the other four clones (NL-pSE, NL-p11807, NL-pDEMB12, and NL-p01TT) did to a lower level (relative levels were 0.50, 0.04, 0.30, and 0.36, respectively). Drastic decrease observed for NL-p11807 could be attributable to an effect by Y226tac variation as described above (**Figures 7**, **10**). These results may suggest that *vif* expression level is mainly determined by some particular nSNV(s) in SA1D2prox, although the clones examined have multiple nSNVs that enhance or reduce *vif* production (**Figure 11**). Somewhat unexpectedly, no clones showed an upregulated expression of *vif* mRNA in the presence of nSNVs with an enhancing effect in SA1D2prox (**Figure 11**). Of proviral clones carrying newly found variations (V234Lctg, D256Egaa, D256Egag, and V259Iata), only V234Lctg significantly increased *vif* expression level (**Figure 12A**).

Nine proviral clones with a naturally occurring SA1D2prox sequence (**Figure 11**) were next examined for their abilities to express Vif protein and *vpr* mRNA for confirmation purpose. As shown in **Figure 12B**, while five clones (NL-p04AR, NL-pCu19, NL-pEC003, NL-pMM45, and NL-pCY165) produced Vif similarly with NL-pC3, NL-p11807 expressed an undetectable level of Vif, being consistent with the RNA data (**Figure 12A**). The other three clones (NL-pSE, NL-pDEMB12, and NL-p01TT) expressed Vif at a lower level relative to that by NL-pC3, again being in agreement with the RNA data (**Figure 12A**). Expression of *vpr* mRNA was then analyzed to ascertain inverse relationship of production level between *vif* and *vpr* RNAs as described above (**Figure 4**). As observed in **Figure 12C**, low *vif*/Vif expressers here (NL-pSE, NL-p11807, NL-pDEMB12, and NL-p01TT) produced higher levels (mean values) of *vpr* mRNA relative to that by NL-pC3, confirming our previous results (Nomaguchi et al., 2016).

**FIGURE 13:**
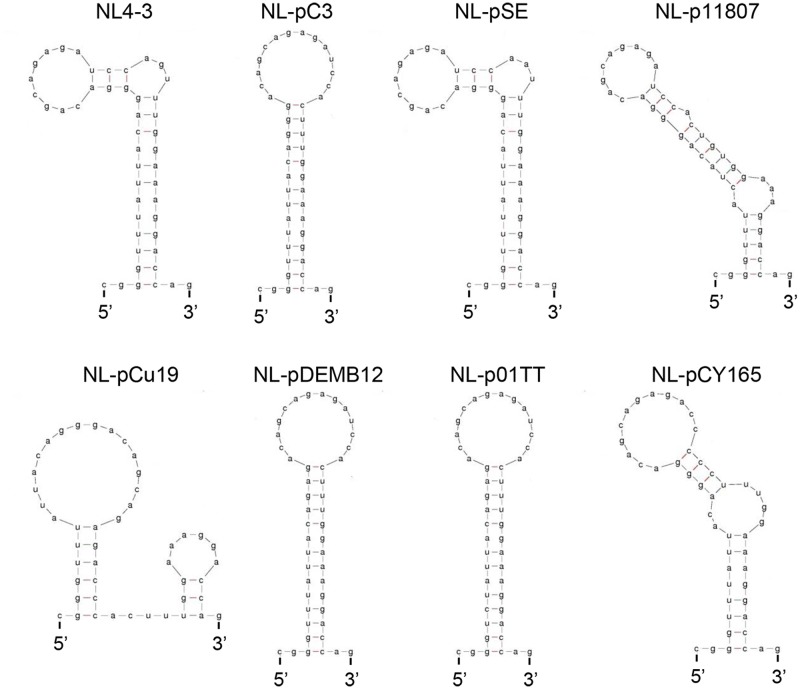
Secondary RNA structures of SLSA1 in naturally occurring full-length SA1D2prox sequences. RNA structural shapes predicted by mfold program (Zuker, 2003) are shown for viral clones in **Figure 11**.

**FIGURE 14:**
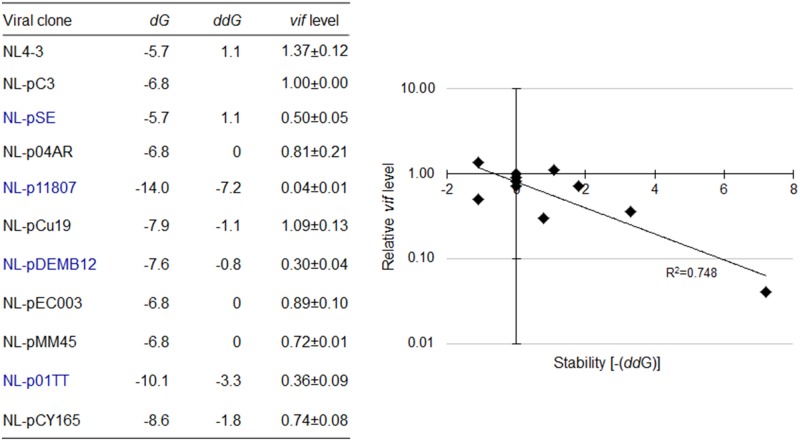
Relationship between *vif* mRNA production and SLSA1 RNA stability for clones carrying naturally occurring full-length SA1D2prox sequences. Free energy (*d*G) for SLSA1 sequence derived from proviral clones indicated were predicted by mfold program (Zuker, 2003). RNA stability (*dd*G) is presented as difference of free energy between NL4-3/each nSNV and NL-pC3. Relative *vif* expression levels to that by NL-pC3 in **Figure 12** were used. Blue letters indicate clones that gave *vif* expression levels significantly lower than that by NL-pC3. Scatter diagram on the right was created by plotting *vif* expression levels and SLSA1 stabilities [–(*dd*G)] of the clones relative to those of NL-pC3. Exponential trendline and coefficient of determination (*R*^2^ = 0.748, *p* = 0.019 by *F*-test) are shown.

We then performed structural (**Figure 13**) and stability (**Figure 14**) analyses of SLSA1 derived from clones with a naturally occurring SA1D2prox (**Figure 11**) as described above (**Figures 6**, **7**, **9**, **10**). Because SLSA1 sequence of three proviral clones (NL-p04AR, NL-pEC003, and NL-pMM45) was identical to that of NL-pC3 (**Figure 11**), we first predicted RNA properties of NL-pC3 SLSA1. As shown in **Figures 13**, **14**, the secondary structure and free energy (*d*G) of NL-pC3 SLSA1 were different from those of NL4-3 due to a G⋅U wobble pair and a G-C canonical pair formed by substitution of V234gtt to L234ctt in the stem. While SLSA1 secondary structures of NL-pDEMB12 and NL-01TT were the same with that of NL-pC3, free energies for both structures were increased by formation of an A-U canonical pair and A-U/C-G canonical pairs, respectively, relative to that for NL-pC3 SLSA1. NL-pSE showed the same SLSA1 structure and free energy with those of NL4-3 SLSA1 despite the presence of a single-nucleotide substitution. SLSA1 secondary structures of the other three clones (NL-p11807, NL-pCu19, and NL-pCY165) were predicted to be unique, and free energies for the structures were different from those for predicted SLSA1 secondary structures of NL-pC3 and NL4-3.

In order to finally determine whether there is some specific relationship between *vif* production and SLSA1 structure, if any, we again created a scattered diagram by plotting *vif* expression levels and SLSA1 stabilities (*dd*G) relative to those by NL-pC3. As seen in **Figure 14**, interestingly and importantly, we found that *vif* production level is inversely correlated with SLSA1 stability upon examination of proviral clones with naturally occurring full-length SA1D2prox sequences from HIV-1 strains (*R*^2^ = 0.748). Even if data for NL-p04AR, NL-pEC003, and NL-pMM45, which carry identical SLSA1 sequence with that of NL-pC3, were excluded from analysis, or, relative data to those by NL4-3, instead of those by NL-pC3, were used for analysis, similar results (*R*^2^ = 0.732 and 0.742, respectively) were obtained. These results are quite contrasting to those in **Figures 7**, **10**, and indicate that there may be a virologically significant association between the Vif production and the SLSA1 structure.

## Discussion

In this study, we investigated effects of nSNVs in a genomic SA1D2prox region on *vif* expression and SLSA1 structure, and resultantly demonstrated functional importance of SA1D2prox for modulation of *vif* mRNA production. Nine nSNVs that significantly affect *vif* expression level (P233ccc, P233ccg, V234gtc, K240aaa, L242ctt, A248gcg, V249gtg, N254aac, and V259gtg) were newly found in SA1D2prox in addition to those previously identified in SA1prox (Nomaguchi et al., 2016) (**Figures 3**, **5**, **7**). Many of the nSNVs identified by us so far were clustered in SLSA1 within SA1D2prox, suggesting a possible role for SLSA1 in *vif* production (**Figure 5**). For proviral clones carrying a single nSNV within SLSA1 and their SLSA1 structural mutants, no clear correlation between *vif* expression levels and changes in secondary structure/stability of SLSA1 was observed (**Figures 7**, **10**). In contrast, for proviral clones with full-length viral genomic SA1D2prox sequences, which contain multiple nSNVs from HIV-1 subtype B strains, a significant inverse correlation between *vif* expression levels and structural stabilities of SLSA1 RNAs was recognized (**Figure 14**). It has been reported that two silent mutations introduced into HIV-1 NL4-3 SLSA1 significantly affect usage of SA1 site (Emery et al., 2017). The observed inverse correlation between *vif* expression levels and SLSA1 stabilities represents the first biological evidence to show the functional importance of SLSA1 secondary structure, revealed by the *in vitro* chemical probing approach without any cellular components. Moreover, our findings have important implications for elucidating the mechanisms by which SLSA1 regulates *vif* expression. *Vif* mRNA is produced by alternative splicing of HIV-1 pre-mRNA. In general, recognition of the splice sites by spliceosome involves interactions between protein factors and a single-stranded portion of the pre-mRNA (Maris et al., 2005; Hiller et al., 2007). Interestingly, however, exon/intron junctions that undergo alternative splicing are often thermodynamically more stable than that of constitutive exons, indicating the formation of RNA secondary structures (Shepard and Hertel, 2008). Such local RNA structures can interfere with spliceosomal assembly through sterical masking of splice sites or enhancer binding sites. Alternatively, such RNA secondary structures can accelerate spliceosomal assembly by shielding splicing repressor binding sites (Hertel, 2008). Present finding of inverse correlation between SLSA1 stabilities and *vif* expression levels suggests that the SLSA1 region may act for masking splice sites or enhancer binding sites for splicing rather than the masking of repressor binding sites. Further study to elucidate tertiary interactions of the SA1D2prox and splicing regulators is necessary to address this issue. Because the splicing at SD1 and SA1 sites is essential for *vif* mRNA production, nSNVs we identified in this study most likely affect the splicing efficiency at SA1 site. Therefore, molecular biological analyses including experiments to determine the splicing efficiency at SA1 site and the splicing regulatory factors associated with the nSNVs are currently in progress in our laboratory.

The fact that a meaningful correlation between *vif* expression level and SLSA1 stability is observed only for proviral clones carrying naturally occurring SA1D2prox is of virological significance (**Figures 11**, **14**). Biological and molecular bases for emergence of such effective SA1D2prox sequences are totally unclear at present, and remain to be clarified. Although unknown for driving forces to generate nSNVs that affect *vif* mRNA production, multiple mutations accumulated in the region over time would benefit HIV-1 through a Vif-mediated replication modulation. Clearly, this is not the case with proviral clones that contain artificially constructed SA1D2prox sequences (**Figures 7**, **10**). Given a current model that biologically relevant RNA structures are evolutionarily retained (Jin et al., 2011), when some mutations introduced into functionally important RNA structures of viruses have biologically adverse effects, it is expected that viruses carrying such mutations are eliminated during replication cycles, or that an RNA structure critical for viral replication is maintained by acquiring another mutation(s) in their genomes. Thus, the introduction of an individual nSNV into HIV-1 NL4-3 in our experiments, even if the nSNV is found in the genomes of circulating or spreading HIV-1 strains, would be a transient and artificial mutation for viruses, and the change in SLSA1 structural shape/stability by the nSNV also may not be preferable for HIV-1. On one hand, recent structure analyses on a full-length RNA genome of HIV-1 NL4-3 have shown that SA1D2prox sequence (excluding SLSA1) can interact with its upstream and downstream regions (Watts et al., 2009; Pollom et al., 2013). For naturally occurring viruses, multiple mutations may have accumulated over time in the entire SA1D2prox region including SLSA1, and thereby have stabilized the whole RNA structure surrounding and containing SA1D2prox. The nSNVs in SA1D2prox may primarily affect *vif* production via various regulatory elements and factors (**Figure 5**) (Kammler et al., 2006; Exline et al., 2008; Mandal et al., 2009; Widera et al., 2013; Brillen et al., 2017). Consequently, we could expect that the importance of SLSA1 structure and SA1D2prox sequence for *vif* production became obvious only for proviral clones carrying natural SA1D2prox sequences. In agreement with previous reports (Pollom et al., 2013; Nomaguchi et al., 2016), both the RNA long-range interaction and the SLSA1 local structure may play a key role in regulating production of HIV-1 mRNAs.

Based on result shown in **Figure 14**, it is likely for naturally occurring HIV-1s that increase in SLSA1 RNA stability leads to decrease in *vif* production. This interpretation indicates that structural stabilization of SLSA1 may limit *vif* production. Watts et al. (2009) reported that, while highly used splicing acceptors are unstructured, SA1 site shows the lowest SHAPE (selective 2′-hydroxyl acylation analyzed by primer extension) reactivity, i.e., a stabilized secondary structure, and is a rarely used acceptor (Purcell and Martin, 1993). Moreover, we and others have shown that excessive Vif expression gives a negative effect on viral replication (Akari et al., 2004; Madsen and Stoltzfus, 2006; Exline et al., 2008; Nomaguchi et al., 2016). Thus, stability of SLSA1 structure in addition to a nearby sequence of SA1 site may serve to suppress surplus usage of SA1 site critical for *vif* mRNA production. In this regard, it has been reported that decrease in SA3 utilization is attributed to increased stability of a stem-loop structure containing SA3 site in the context of polypyrimidine tract sequence (Jacquenet et al., 2001). This suggests that the SA3 usage is regulated not only by the stability of the stem-loop structure but also by the sequence of a region around SA3 site, being consistent with modulation of *vif* expression by both the structural stability of SLSA1 and the SA1D2prox sequence. It has been reported that SD1 site is also included in a semistable hairpin structure (Abbink and Berkhout, 2008). Stabilization of SD1 hairpin structure by introducing mutations has been shown to cause severe defects in viral replication through effects on the splicing at SD1 site (Abbink and Berkhout, 2008). Totally, it is not unreasonable to assume that both the SA1D2prox sequence and the stability of SLSA1 structure influence *vif* production via effects on SA1 usage.

Expression levels of *vif* mRNA and Vif protein by nine tested proviral clones with naturally occurring entire SA1D2prox were not higher than those by NL4-3 and NL-pC3 (**Figure 12**). Rather, some clones (NL-pSE, NL-p11807, NL-pDEMB12, and NL-p01TT) produced reduced levels relative to NL4-3 and NL-pC3. As described previously (Nomaguchi et al., 2016), decrease in *vif* production is not always disadvantageous to HIV-1, unless the power balance between Vif and APOBEC3G/F is completely disrupted. Restrictive effects imposed by a sub-lethal level of APOBEC3G/F can rather be advantageous to HIV-1 for survival, because HIV-1 may readily acquire mutations under such circumstances to increase genome variations. This is beneficial for HIV-1 to adapt and evade from unfavorable environments such as host immunity and medication (Mulder et al., 2008; Jern et al., 2009; Sadler et al., 2010; Kim et al., 2014). Indeed, while the growth capacity of Y226tac virus (low *vif* type in **Figures 7**, **10**) is markedly restricted in H9 cells with a high APOBEC3G expression level (Nomaguchi et al., 2016), adapted viruses emerged from a long-term culture of H9 cells initially infected with a high dose of this virus clone. Sequence analysis of the adapted viruses showed that numerous mutations are present throughout viral genomes. Of these, a growth-enhancing mutation that moderately restores *vif* expression level was identified within the Vif-coding sequence located downstream of SA1D2prox. These results demonstrate a high potential of HIV-1 to adapt and survive under strictly replication-restrictive conditions (our unpublished data). These results also indicate that *vif* expression level can be modulated by sequences other than SA1D2prox. Presence of many elements/sites involving in modulation of *vif* production is suggested by the effect of G_I3_-1 element in the Vpr-coding sequence on *vif* production, and by inverse correlation between *vif* and *vpr* expression levels (Widera et al., 2014; Nomaguchi et al., 2016) (**Figures 4**, **12**). We and others have consistently shown that viral mRNAs encoding for Vif and Vpr are expressed in a mutually exclusive manner (Widera et al., 2014; Nomaguchi et al., 2016). However, molecular bases for this relationship remain elusive, and further study is needed to elucidate its underlying mechanism.

## Conclusion

Since Vif is an essential factor for virion infectivity, elucidating the sequence and RNA structure relevant to the modulation of *vif* production is useful to establish systems for effective control of HIV-1 replication. Moreover, a better biological understanding of local RNA structures and RNA long-range interactions in HIV-1 and the other viral RNA genomes would contribute to the progress in RNA biology.

## Author Contributions

MN designed the research, performed the experiments, discussed the results, and wrote the manuscript. ND, TY, and TK performed the experiments and discussed the results. SA performed the statistical analysis. HO and YI performed the sequence analysis and discussed the results. MY and HS discussed the results. AA designed the research, discussed the results, and wrote the manuscript.

## Conflict of Interest Statement

The authors declare that the research was conducted in the absence of any commercial or financial relationships that could be construed as a potential conflict of interest. The handling Editor declared a shared affiliation, though no other collaboration, with several of the authors MY and HS.

## Footnotes

1 http://www.hiv.lanl.gov/

2 http://weblogo.threeplusone.com/

3 http://unafold.rna.albany.edu/?q=mfold/RNA-Folding-Form

4 http://www.hiv.lanl.gov/
